# Comprehensive Analysis of Rheological, Mechanical, and Thermal Properties in Poly(lactic acid)/Oxidized Graphite Composites: Exploring the Effect of Heat Treatment on Elastic Modulus

**DOI:** 10.3390/polym16030431

**Published:** 2024-02-04

**Authors:** Mónica Elvira Mendoza-Duarte, Alejandro Vega-Rios

**Affiliations:** Centro de Investigación en Materiales Avanzados, S.C. (CIMAV), Av. Miguel de Cervantes #120, Complejo Industrial Chihuahua, Chihuahua 31136, Mexico

**Keywords:** PLA, expanded graphite, mechanical properties, crystallinity grade

## Abstract

This study is focused on investigating the rheological and mechanical properties of highly oxidized graphite (GrO) incorporated into a poly (lactic acid) (PLA) matrix composite. Furthermore, the samples were annealed at 110 °C for 30 min to study whether GrO concentration has an effect on the elastic modulus (E’) after treatment. The incorporation of GrO into PLA was carried out by employing an internal mixing chamber at 190 °C. Six formulations were prepared with GrO concentrations of 0, 0.1, 0.5, 1, 1.5, and 3 wt%. The thermal stability, thermomechanical behavior, and crystallinity of the composites were evaluated utilizing thermogravimetric analysis (TGA), dynamic mechanical analysis (DMA), and differential scanning calorimetry DSC, respectively. The thermal stability (according to T_max_) of the PLA/GrO composites did not change substantially compared with PLA. According to DSC, the crystallinity increased until the GrO concentration reached 1 wt% and afterward decreased. Regarding the heat treatment of the PLA/GrO composites, the E’ increased (by two orders of magnitude) at 80 °C with the maximum value achieved at 1 wt% GrO compared with the non-heat-treated composites.

## 1. Introduction

The degradation of polymers is a phenomenon that depends on different and numerous factors, such as humidity, temperature, light, and the presence of enzymes or impurities [1,2]. In addition, the thermal and mechanical stability and other properties of the polymers can be modified by the incorporation of substances or polymers [3], additives [4], reinforcements [5], nanoparticles [2], and clays [6]. Poly(lactic acid) (PLA) is a biodegradable aliphatic polyester derived from agricultural plants such as corn, sugar beets, and wheat, among others [7]. Furthermore, PLA is the most studied biodegradable polymer among many polyesters, with the advantages of high strength and a high modulus. It can be processed using conventional techniques for thermoplastic polymers, such as injection, blow molding, thermoforming, and extrusion [8]. PLA’s applications have mainly been in biomedical, pharmaceutical, and automotive devices [9].

Additionally, PLA is recommended by the United States Food and Drug Administration (FDA) for its use in the production of food containers [10,11], and it could be an excellent alternative to white pollution [12]. It is stable under typical use conditions, retaining its molecular weight and physical properties for years [13]. However, in a specific application, there is a natural concern regarding the durability of polymeric materials in terms of their useful lifetime and rapid degradation. The degradation reaction of PLA can be modified by adding different substances or fillers [14,15].

On the other hand, graphene is a nanofiller with the potential to enhance the properties of neat polymers [10,16]. It is a single sheet of carbon with a two-dimensional honeycomb structure [14,17] in which carbons are bonded together and possess a vast surface area (2600 m^2^/g) [1,18]. Compared with pristine graphene, graphene oxide is more heavily oxygenated and has a hydrophilic nature due to its structure and characteristic functional groups (hydroxyl, epoxy, carbonyl, and carboxyl) [1,14,19,20,21].

Diverse works pinpoint that the addition of graphene oxide (GO) or reduced graphene oxide (rGO) to a polymer matrix makes it possible to control the degradation behavior of the polymer, accelerating or postponing the degradation process. For example, Jafari et al. [22] estimated the influence of GO on the hydrolytic degradation of polyethylene terephthalate (PET)/PLA/GO blends. Their results showed that with the addition of 1 wt% GO, the hydrolytic degradation of the blend was intensified compared with that of the neat blend. Verma et al. [23] studied the photocatalytic degradation of polypropylene (PP)/rGO/titanium dioxide (TiO_2_) nanocomposites and observed that the interaction of rGO/TiO_2_ nanoparticles enhanced the photocatalytic degradation of PP. Likewise, Jeong et al. [14] prepared PLA/lipophilized GO (LGO) nanohybrids by the solution blending method and observed that the presence of GO functionalized with octadecylamine reduced the hydrolytic degradation of PLA.

The incorporation of zinc oxide (ZnO) nanoparticles is an additional example, which is not recommended at concentrations higher than 0.1 wt% because they affect the absorption of UV light, the glass transition temperature (T_g_), and thermal degradation [24]. The transesterification reactions and interactions decreased between components of the PLA/aluminum diethylphosphinate composite when using silicon dioxide (SiO_2_) and halloysite particles. Only the organically modified montmorillonite presents improved thermal stability and relative thermal degradation yields in the PLA/aluminum diethylphosphinate composite [25]. Yang-Bing et al. [26] found that the PLA degradation rate was accelerated when TiO_2_ particles were added, reporting a decrease in PLA’s molecular weight of 30% when 15 wt% TiO_2_ was added. They also found that the crystallinity of the composite was modified when the PLA degradation took place.

The main parameters used to analyze the degradation process in a polymer include the loss of mechanical strength of the polymer matrix and the reduction in the molecular weight. In particular, this latter factor can be evaluated by employing viscosity measurements. In addition, the crystallinity of PLA tends to increase as it degrades because the chain scissions mainly occur in the amorphous regions, increasing the global crystallinity of the polymer [27]. In order to understand the effect of crystallinity on the mechanical, dynamic mechanical, and thermal properties of PLA, a few researchers have conducted comparisons of PLA’s properties, specifically those of PLA/Talc, before and after applying different heating treatments and found that the presence of crystals improves the thermomechanical properties [28,29].

Despite the fact that other studies on PLA/GO composites have been conducted, they do not fully investigate the rheological properties of the composites to study the formed structure, morphology, and interactions. In the same way, the effect of a heat treatment on the elastic modulus (E’) has been addressed.

Herein, the aim of this study was to evaluate the effects of the inclusion of graphite oxide (GrO) on the rheological, thermal, and mechanical properties of PLA, especially the E’, after applying a heat treatment on PLA/GrO composites. Thus, these materials were characterized by rotational rheometry, dynamic mechanical analysis (DMA), thermogravimetric analysis (TGA), differential scanning calorimetry (DSC), transmission electron microscopy (TEM), and field-emission scanning electron microscopy (FE-SEM).

## 2. Materials and Methods

### 2.1. Materials

Poly (L,L-lactide) (2002D) (PLA), supplied by NatureWorks LLC (Minneapolis, MN, USA), had the following characteristics: specific gravity = 1.24, melt index = 4–8 g/10 min (190 °C). As a precursor for graphite oxide (GrO), natural graphite flakes (325 mesh, 99.8% (metals basis) Alfa Aesar) were employed, hereafter called G325. Sulfuric acid (H_2_SO_4_), sodium nitrate (NaNO_3_), hydrogen peroxide (H_2_O_2_), and potassium permanganate (KMnO_4_) were purchased from Sigma-Aldrich (San Luis, MO, USA). All the chemicals were of analytical reagent grade and used as received without further purifications.

### 2.2. Graphite Oxidation

GrO was obtained from G325 using a modified Hummer’s method approach [30]. The oxidation process consisted of mixing G325 (15 g) and NaNO_3_ (7.5 g), followed by the addition of H_2_SO_4_ concentrated (345 mL) under stirring at a constant temperature of 0–4 °C. Later, KMnO_4_ (45 g) was added slowly under steady stirring and controlled at 20 °C to prevent overheating and explosion. Next, the dispersion was stirred and heated until reaching a temperature of 35 °C. Finally, to ensure the completion of the reaction with KMnO_4_, the solution was diluted by adding distilled water (690 mL) and 3% H_2_O_2_ solution (600 mL). The resulting solution was centrifuged and washed several times until it reached a pH of 6. Afterward, the dispersion was sonicated for 15 min and dried at 60 °C for 24 h to ensure complete water elimination. The dried product was a film and was milled for 15 min in a blade grinder, BEL-ART.

### 2.3. Preparation of Composites

PLA/GrO composites were obtained via melt blending in a Brabender Plasti-corder mixing machine (DDRV50, C.W. Brabender Instruments Inc., Hackensack, NJ, USA) using CAM-type blades (which apply moderate mixing) [31]. The mixing procedure was divided into 3 stages. In stage 1, the PLA was premixed at a speed of 30 rpm for 2 min; the torque is not increased during its incorporation into the chamber. In stage 2, an amount of GrO was added according to the formulation of the composite material. In stage 3, the composition was mixed in the chamber at a speed of 90 rpm for 10 min. The total mixing time was 12 min for all formulations. The mixing temperature was set at 190 °C. The weight fractions of GrO were fixed at 0, 0.1, 0.5, 1.0, 1.5, and 3 wt% and were called neat PLA, PLA/0.1GrO, PLA/0.5GrO, PLA/1GrO, PLA/1.5GrO, and PLA/3GrO, respectively.

### 2.4. Film Preparation

Films for characterizations and measurements were developed by hot-press molding (Carver Inc., Wabash, IN, USA). Firstly, the composites obtained were ground in a blade grinder (FRITSCH Pulverisette, Idar-Oberstein, Germany). Next, the ground formulation was placed in a mold at 190 °C for 3 min, with no pressure. After this, the pressure was increased to 4 tons for 2 min. Finally, the films were cooled in a water bath at a constant temperature of 13 °C for 5 min. The thickness of PLA/GO films was 0.15 mm. Probes were cut to the appropriate dimensions for characterizations.

### 2.5. Characterization

#### 2.5.1. Rheological Properties

The effect of GrO on the rheological properties of the PLA matrix was evaluated in a rotational rheometer Physica model MCR 501 (Anton Paar, Graz, Austria) employing a parallel-plate geometry. The dynamic complex viscosity (η*), storage modulus (G′), and loss modulus (G″) were measured at 190 °C as a function of angular frequency (ω) ranging from 0.1 to 100 rad/s. The linear viscoelastic region (LVR) of the composites was determined by means of a strain sweep from 0.001% to 1000% at a frequency of 1 Hz.

#### 2.5.2. Dynamic Mechanical Analysis

DMA was carried out using a DMA RSAIII (TA Instruments, New Castle, DE, USA) in a tensile configuration. To determine the thermomechanical behavior of the samples (3.0 mm × 0.25 mm × 20 mm), a temperature ramp was performed from 25 °C to 140 °C. The deformation frequency was fixed at 1 Hz, and the applied deformation percentage was 0.1%. To evaluate if GrO could promote the formation of crystals due to cold crystallization and, thus, impact the thermomechanical properties of the composites, a time sweep was performed at 110 °C for 30 min, simulating a “heat treatment”. The heat treatment effect was evaluated using DMA with a temperature ramp over the heated samples.

#### 2.5.3. Thermogravimetric Analysis

The thermal stability of the PLA/GrO composites and the graphite powders was determined using a TGA Q600 (TA Instruments, New Castle, DE, USA). The samples (13.03 ± 0.05 mg) were heated from room temperature to 800 °C with a heating ramp of 10 °C/min under airflow. In addition, to evaluate if the GrO experiments showed some changes in its oxidation grade during the composite compounding, GrO was analyzed by TGA in an inert atmosphere, applying an isotherm at 190 °C for 10 min (simulating the heating that GrO experiments during processing); after that, a temperature ramp was used at a heating rate of 5 °C/min.

#### 2.5.4. Differential Scanning Calorimetry

In order to evaluate if any changes in the PLA structure occurred with the addition of GrO, DSC was employed. DSC measurements were performed using a DSC Q2920 (TA Instruments, New Castle, DE, USA) under a static atmosphere utilizing aluminum pans. The heating rate was 10 °C/min from 30 °C to 200 °C. The thermal history of samples was erased by a preliminary heating–cooling cycle at 10 °C/min. The glass transition temperature (T_g_), cold crystallization temperature (T_cc_), melting temperature (T_m_), and cold crystallization enthalpy (ΔH_cc_) were determined from the second scan at 10 °C/min.

#### 2.5.5. Electron Microscopy

The surface morphology of G325 and GrO was performed over carbon-coated copper grids. The surface morphology analysis and element content (carbon and oxygen) were obtained by field-emission scanning electron microscopy (FE-SEM) (JEOL JSM-7401F, Tokyo, Japan) with a 2 keV acceleration. The dispersion of GrO into the PLA matrix was characterized by transmission electron microscopy (TEM). PLA/GrO composites were microtomed (Reichert Ultracut, Eindhoven, The Nederland) at room temperature into 70 nm–90 nm thick slices with a diamond knife and placed onto 300-mesh Cu grids. Micrographs were collected using a transmission electron microscope (TEM, Hitachi 7700, Tokyo, Japan) at an accelerating voltage of 60 kV. High-resolution micrographs were performed TEM (Jeol JEM 2200 FS+CS, Tokyo, Japan).

#### 2.5.6. Fourier Transform Infrared Spectroscopy

The FTIR powders and composites were measured using an IR Affinity 1S B spectrometer (Shimadzu, Kyoto, Japan). Spectra were obtained by reflectance employing a Total Attenuated Reflectance accessory, Specac model Quest. The samples were analyzed in transmittance mode in the range 600 cm^−1^–4000 cm^−1^ with a resolution of 4 cm^−1^.

#### 2.5.7. Raman Spectroscopy

Raman spectra of G325 and GrO were obtained using a LabRam HR Vis-633 with a HeNe 632.8 nm laser (Horiba, Kyoto, Japan). The Raman data acquisition took place from 100 cm^−1^ to 3500 cm^−1^ at room temperature.

#### 2.5.8. X-ray Diffraction

XRD measurements of powders were carried out on a Bruker D8 ADVANCE (Bruker Corp., Billerica, MA, USA) with CuKα radiation (λ = 1.54 A°). Data were collected from 4° to 70° at a scan rate of 3.3 min^−1^.

#### 2.5.9. X-ray Photoelectron Spectroscopy

XPS of G325, GrO, and GrOmb (GrO treated under melt-blended conditions) was performed at a temperature of 25 °C on an ESCALAB 250 Xi (Thermo Scientific, Paisley, UK) using Kα excitation radiation (hm = 1486.6 eV). The pass energy was set at 4.2 eV. The angle employed was 45°, and the vacuum pressure of analysis was ~10–8 mbar. To evaluate if the temperature affected GrO functional groups during processing, GrO was heated for 10 min at 190 °C, and XPS data were acquired.

#### 2.5.10. Energy-Dispersive Spectra (EDS)

Elemental analysis of GrO and G325 was performed in an electron microscope, HITACHI 3500 (Santa Clara, CA, USA), equipped with an energy-dispersive X-ray spectroscope (EDS). Powders were placed onto 300-mesh Cu grids for analysis.

#### 2.5.11. Dynamic Light Scattering (DLS)

The particle size of the GrO was determined by DLS (MasterSizer 2000, Malvern Instruments, Malvern, Worcestershire, UK). The dispersion of GrO (1 wt%) was sonicated prior to analysis.

## 3. Results and Discussion

The G325 and GrO characterization results are illustrated in the supplementary material, specifically Figures S1–S6 and Table S1. The particles of GrO used in the experiments had an average size of 323.5 μm. The obtained GrO had an oxygen content of 40 At % (Table S1). It is important to note that only the results and discussion for PLA/GrO composites are presented below.

### 3.1. Rheology

**Figure 1:**
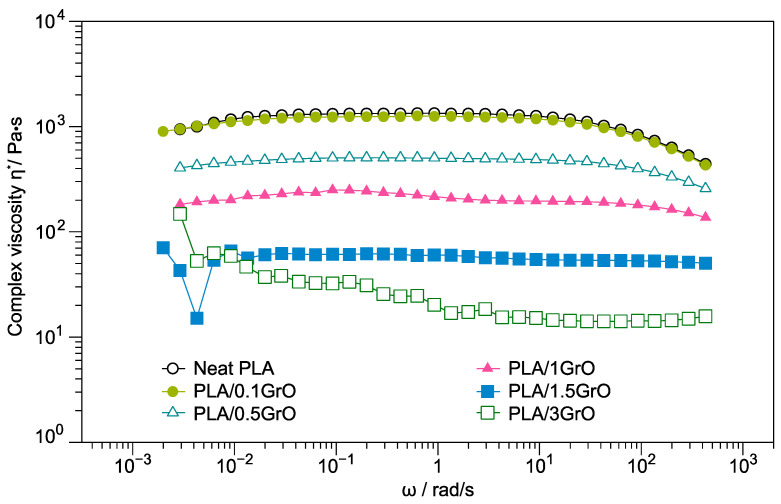
Complex viscosity of neat PLA and PLA/GrO composites (PLA/0.1 GrO, PLA/0.5 GrO, PLA/1GrO, PLA/1.5 GrO, PLA/3GrO).

The complex viscosities (η*) of the neat PLA and PLA/GrO composites are shown in Figure 1, which were studied at a temperature of 190 °C. The samples of neat PLA, PLA/0.1GrO, PLA/0.5GrO, and PLA/1GrO at low frequencies presented a plateau. However, they presented a shear thinning behavior at high frequencies, behaving like a non-Newtonian fluid [32,33].

In general, the viscosity of the PLA/GrO composites decreased with increasing GrO concentration in the polymer matrix, which can possibly be attributed to an associated rise in the mobility of the polymeric chains in the molten state [34,35]. Several theories could explain the observed behavior. First, the molecular weight of PLA diminished due to the degradation of the PLA matrix. The large number of oxygen-containing functional groups in GrO promotes the hydrolysis of ester bonds of the PLA. This reaction is caused by the attack of water molecules on PLA, accelerating its degradation. It has also been reported [36] that the presence of hydroxyl groups can favor alcoholysis reactions. These facts could explain the lower viscosity of the composite as the GrO content increased, which can be attributed to the breakdown of PLA’s molecular chains [37], causing higher losses of molecular weight compared with the unfilled PLA [38].

Moreover, the second theory considers the lubricant effect, where GrO layers slide during the hot-melt viscosity measurements of PLA/GrO blends, acting as a plasticizer [34,39]. Therefore, this movement increases with GrO concentration in the PLA matrix. Finally, another possibility is that the oxidation reaction results in an increase in the GrO interplane space, which decreases the intermolecular forces compared with graphite [40]. For this reason, at low frequencies, an increase in the complex viscosity of the PLA/1.5GrO and PLA/3GrO composites was observed, indicative of filler–polymer and filler–filler interactions [34,41,42].

On the other hand, similar viscosity behavior was observed by Cho and Paul [43] when studying Nylon 6 composites with three different fillers (glass fiber, montmorillonite, and organoclay) measured in a capillary rheometer. They suggest that the viscosity reduction is due to two possible reasons: the first one is the glissades between the polymer matrix and the exfoliated organoclay at high shear flow, and the second is the molecular weight decrease in Nylon 6 owing to its degradation (hydrolysis).

**Figure 2:**
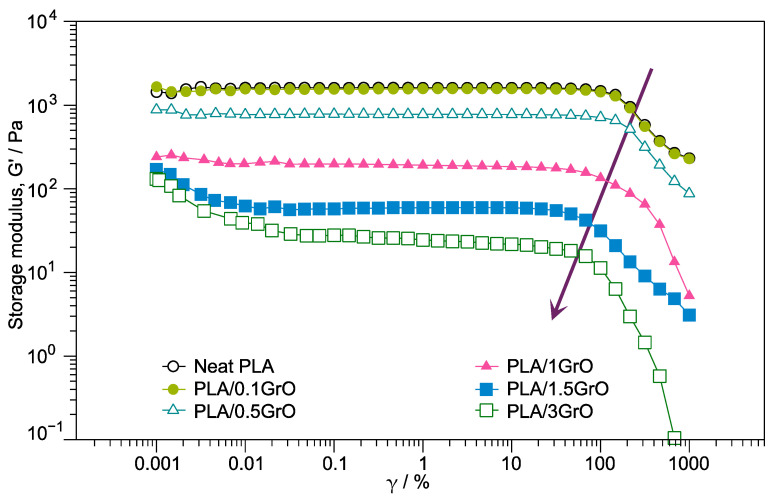
G′ as a function of γ of neat PLA and PLA/GrO composites (PLA/0.1 GrO, PLA/0.5 GrO, PLA/1GrO, PLA/1.5 GrO, PLA/3GrO). The arrow indicates the direction in which the concentration of GrO increases.

Figure 2 displays the G′ of the PLA/GrO composites exhibited at low strains on a Newtonian plateau and a non-linear region at high strain amplitudes. As can be observed, the structural properties became weaker at lower strain as the GrO content increased. In contrast, the decrease in G′ at high strains was due to the change in the structural properties [33,44].

Compared with the neat PLA, PLA/GrO composites generally had lower G′ values with increasing GrO concentration. Durmus and Macosko [45] observed this behavior in oxidized polyethylene (OxPE) composites filled with organoclays at 1 wt% and 2 wt%. They attributed the reduction in the G′ value to the effects of the low molecular weight and low melt viscosity of OxPE. Another theory is the exfoliation of the GrO layers and the formation of more fragile filler networks [46]. In addition, an analysis in the linear viscoelastic region (LVR) indicated a decrease depending on the increase in GrO concentration in the composites. For example, when analyzing PLA/3GrO, the sample with higher GrO content, the delamination of the GrO layers was found to significantly affect the elasticity of the composite because the GrO stacks are more rigid than exfoliated.

**Figure 3:**
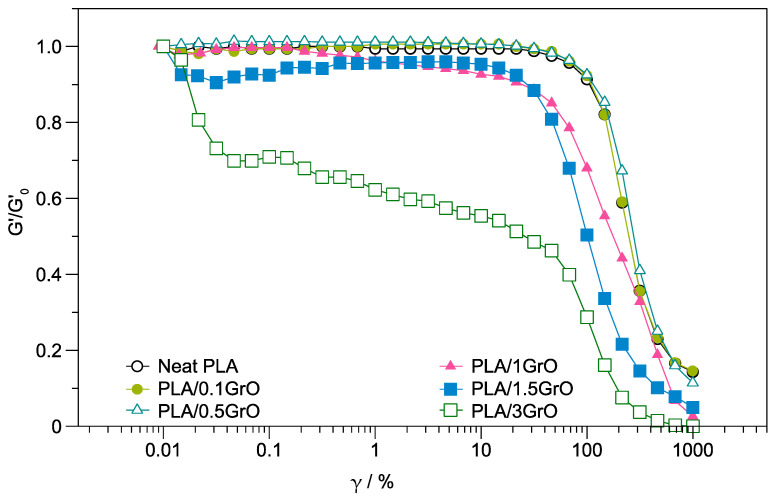
Normalized (G′/G′_0_) vs. γ for neat PLA and PLA/GO composites.

The normalized G′ vs. γ values at a frequency of 1 Hz for neat PLA and PLA/GrO composites are plotted in Figure 3. The results show that the deviation from the linear behavior for PLA/GrO composites occurred at GrO concentrations higher than 0.5 wt%. The critical strain, indicated by a line at the point where G′ diminishes by 5% (G′/G′_0_ = 0.95), was almost the same for neat PLA and composites with 0.1 wt% and 0.5 wt% GrO: γ_crit_ = 70%. The samples PLA/1GrO, PLA/1.5GrO, and PLA/3GrO presented a strain amplitude dependent of the normalized modulus during the shear thinning regime increases. Other authors observed identical behavior in polymer/layered silicate nanocomposites [47,48].

**Figure 4:**
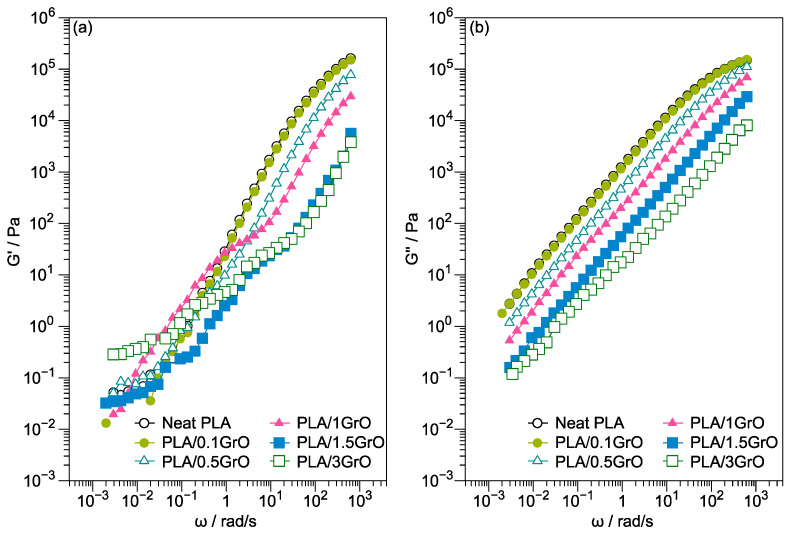
(**a**) G′ and (**b**) G″ of neat PLA and PLA/GO composites.

Figure 4 displays the G′ and G′’ of the neat PLA and PLA/GrO composites at different frequencies. Figure 4a shows a frequency-dependent behavior at high frequencies for G′, and as the GrO content in the PLA matrix increased, G′ decreased. This behavior is opposite to what is expected when a reinforcing filler is added to a polymer matrix. Identical behavior has been previously observed in different PLA blends and composites [4,34], where a decrease in G′ with the increase in filler content is explained by a plasticizing effect imparted by the filler.

Nonetheless, in PLA/GrO composites, the G′ values decreased regarding the concentration attributed to PLA degradation by hydrolysis caused by the liberation of oxygen during the production of the PLA/GrO composites. On the other hand, in the case of matrixes susceptible to hydrolysis, when including PLA or fillers that can act as lubricants, e.g., GrO, the opposite can occur. Rizvi and coworkers [49] observed identical viscoelastic behavior in composites of PLA-Chitin. They attributed this to a combined effect of the presence of larger chitin aggregates and the hydrolytic degradation of PLA at chitin concentrations below 2 wt%. Furthermore, the higher concentration (>2 wt%) of chitin particles resulted in an increase in G′ owing to an enhancement in the reinforcement capacity. They also studied the influence of the addition of maleic anhydride (MA) to the PLA, and the same behavior was observed. Nevertheless, the decrease in G′ was due to the MA free radical grafting, which can cause partial PLA chain scission.

At low frequencies, for neat PLA, PLA/0.1GrO, PLA/0.5GrO, and PLA/1GrO, the PLA chains were relaxed, presenting a terminal behavior (G′ ≈ ω^2^) [33]. In the case of PLA/1.5GrO and PLA/3GrO composites, G′ became independent of the frequency, and the terminal behavior disappeared due to the change in slope observed at high frequencies to a more flattened response at low frequencies, which is associated with a “pseudo solid-like” response of layered nanocomposite melts. The above indicates the formation of an interconnected network of filler–filler contacts in the polymer matrix [33,46,50,51].

Differences in rheological behavior were more significantly pronounced in the case of G´ than G′′. For the G′′ of PLA/GrO composites, Figure 4b shows a slope similar to that of neat PLA at all frequency ranges, and all G′ values of the composites were lower than those of the matrix. A similar rheological response at low frequencies has been observed in composites of PLA and TiO_2_, with the plateau located at these frequencies being attributed to the apparition of a network structure of fillers in the composites [52].

### 3.2. X-ray Photoelectron Spectroscopy

**Figure 5:**
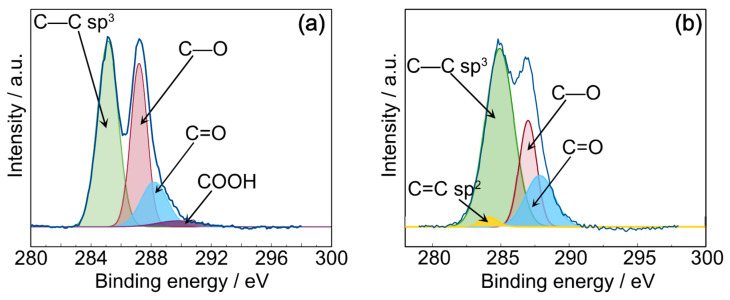
C_1_(s) XPS spectra of (**a**) GrO and (**b**) GrOmb (GrO treated under melt-blended conditions).

The results observed in rheology can be explained by the structural changes in GrO during melt blending. Therefore, GrO was heated at 190 °C for 10 min, simulating the processing conditions of the composites, and analyzed again by XPS and energy-dispersive spectra (Table 1). Figure 5 compares the XPS C1s (284.8 eV) fitted spectra of GrO and GrO treated under melt-blended conditions (GrOmb). Compared with the GrO spectrum, GrOmb modified the proportions of the functional groups. For example, the appearance of the C=C sp^2^ bond, an increase in the C-C sp^3^ and C=O bond, a diminishing C-O bond, and the absence of the -COOH functional group were observed; see Table 1. The C/O ratios for GrO and GrOmb were 2.4 and 2.36, respectively. In summary, this rearrangement of functional groups could cause more significant degradation of the PLA matrix due to an increased hydrolysis reaction.

### 3.3. Dynamic Mechanical Analysis

**Figure 6:**
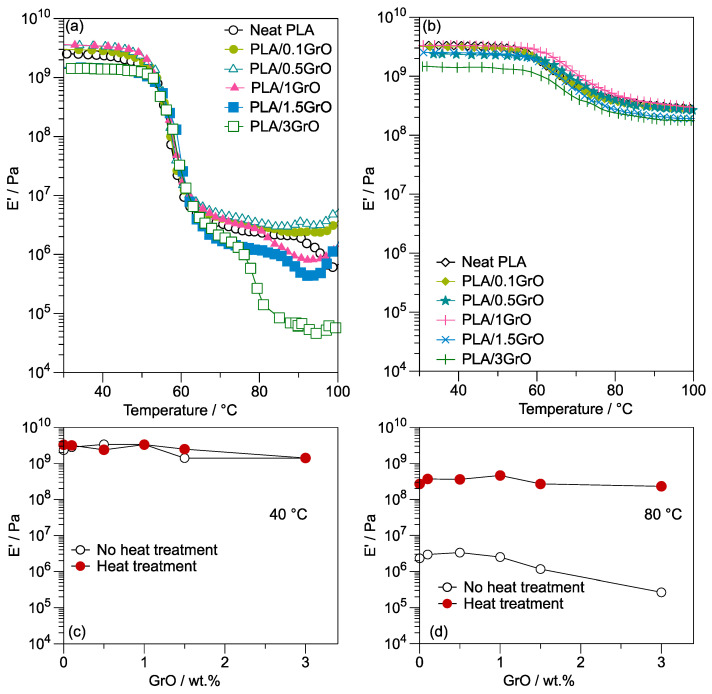
(**a**) E′ of neat PLA and PLA/GO composites as a function of temperature without heat treatment; (**b**) E′ of neat PLA and PLA/GO composites as a function of temperature with heat treatment; (**c**) comparison of E′ at 40 °C; (**d**) comparison of E′ at 80 °C.

DMA showed the motion of the polymer molecules in the glassy/rubbery state. Figure 6a displays the E’ of neat PLA and PLA/GrO composites as a function of temperature. Compared with neat PLA, specifically in the glassy state, PLA/0.1GrO, PLA/0.5GrO, and PLA/1GrO exhibited a rise in E’ at temperatures below the glass transition temperature (T_g_ ≈ 56 °C), indicating that they have a slightly higher stiffness and possess a higher capacity to store energy [34]. In contrast, when the GrO concentration was more than 1 wt%, E diminished, suggesting the formation of a filler network at high concentrations, thus weakening the material. For instance, at a temperature of 30 °C, E’ was reduced from 2.5 × 10^9^ Pa for neat PLA to 1.5 × 10^9^ Pa for the PLA/1.5GrO and PLA/3GrO composites, which corresponds to a decrease of 40%. In the case of other PLA-filler systems, e.g., PLA/Talc, E’ at 30 °C was improved for concentrations higher than 5 wt%. Nevertheless, at higher temperatures, around 100 °C, E′ increased at low talc concentrations (i.e., 1 wt%) [28], indicating that talc acts as a reinforcer. Hence, E′ is not only influenced by the filler content. Ferreira and Andrade [53] attribute this behavior to the tendency of graphene oxide to diminish the crystallinity of PLA.

On the other hand, the processing method and the direction of cutting for the evaluated samples influence the mechanical properties of plastic. Erk et al. [54] estimated E′ at 25 °C in parallel and perpendicular cut specimens obtained from thermoformed plastic cups made of different polymer materials. They reported that a clear polypropylene (PP) cup evaluated in a parallel direction has an E′ value of 1.3 × 10^9^ Pa. In contrast, when measured in a perpendicular direction, the E′ value was 0.67 × 10^9^ Pa. In the case of cups made of polystyrene (PS), the reported values are 0.61 × 10^9^ Pa and 0.79 × 10^9^ Pa in the parallel and perpendicular directions, respectively. Finally, poly (ethylene terephthalate) (PETE) cups had the highest E′ with values of 2.2 × 10^9^ Pa in the parallel direction and 1.2 × 10^9^ Pa in the perpendicular direction.

All composites exhibited a rapid drop in E′ between 60 ° C and 100 °C, i.e., a rubbery plateau. The molecular weight of PLA determines the duration of the initial rubbery plateau and could be considered an indicator of the extent of PLA degradation [55]. Furthermore, the decrease in E′ is attributed to an energy dissipation phenomenon related to the collaborative movements of the polymer chains [56] favored by the presence of GrO. The PLA/plasticizers (i.e., epoxidized palm oil (EPO)) [56] presented a decrease in the E′ with the addition of amounts as low as 1 wt%, indicating an increase in the flexibility of PLA imparted by the EPO. The E′ decreased after the T_g_ because of the softening of the matrix, followed by a horizontal plane provided by EPO related with a slight toughening and elastomeric effect, as observed in our PLA/GrO composite (Figure 6a).

The degree of crystallinity of a material is also related to the rubbery plateau stage. In addition, the formation of α and α’ crystallites formed during the cold crystallization of PLA caused E′ to increase to nearly 100 °C, which implies stiffer composites [57,58,59]. Hence, to evaluate the influence of GrO on the E′ when cold crystallization occurs, the composites were annealed by applying a time sweep at 110 °C for 30 min (Figure 6b). The decrease in E′ above T_g_ was minimized due to crystal formation during heating; see Figure S7. When heated above the T_g_, the polymer chains acquired sufficient mobility in the composites. The E′ values at 40 °C (Figure 6c), the glassy state, were very similar between composites with or without heat treatment. Nevertheless, the E′ values at 80 °C were augmented considerably in annealed samples when experimenting with an increase of around two orders of magnitude; see Figure 6d. At E′_80°C_, there were no significant differences regarding GrO concentration in the composites. However, the PLA/1GrO composite showed a slightly higher E′ with a value of 4.62 × 10^8^ Pa.

**Figure 7:**
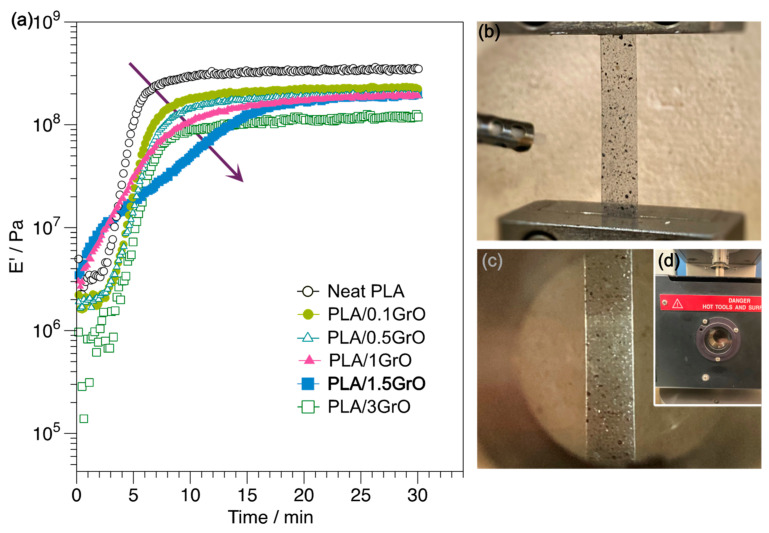
(**a**) Time sweep curve of neat PLA and PLA/GO composites at 110 °C; (**b**) evaluation of thermomechanical properties of neat PLA and PLA/GrO composites from 25 °C to 140 °C. (**c**) Heat treatment of neat PLA and PLA/GrO composites using DMA at 110 °C for 30 min. (**d**) DMA heating chamber.

Moreover, a possible explanation is that the GrO particles acted as nucleating agents at low concentrations, causing the formation of crystals with an increase in E′. The thermodynamic potential and kinetics are the two factors associated with crystal formation. Figure 7a displays the time sweep curve of neat PLA and PLA/GO composites treated at 110 °C for 30 min. Compared with those of neat PLA, PLA/GrO composite curves tend to show a decrease in E′ as a function of increased GrO concentration, except for PLA/3GrO. The time required to reach a stable E′ decreased with an increasing concentration of GrO in the PLA matrix (as indicated by the arrow in Figure 7a), suggesting that each composite exhibits different crystal formation kinetics. For this reason, the designed method of heat treatment lasted 30 min, allowing every sample to achieve a stable E′; see Figure 7b–d. According to the findings for E′ (Figure 6 and Figure 7), GrO acts as a nucleating agent below a concentration of 1 wt% in the PLA/GrO composites.

The incorporation of a second phase into the polymer matrix generally impacts crystallization in two ways: by providing a heterogeneous surface for nucleation and by restricting mobility due to the inclusion of the second phase via interactions of the surface of the particles with the polymer chains, contributing to their immobilization [60,61]. The decrease in E′ (Figure 6b), especially for PLA/1.5GrO and PLA/3GrO, can be attributed to a restriction of the molecular mobility, hindering the crystal formation due to the high GrO concentrations and improved interactions between the filler and the matrix [34,62]. Even when the system’s state is auspicious for crystal formation, slow kinetics can result in a delayed crystallization process.

Moreover, to prevent the product’s deformation or failure, e.g., in food packaging, at high temperatures, it is necessary to have a high E′ to ensure appropriate structural integrity and stiffness. A high E′ (which can be achieved by adding different fillers or nucleating agents) is also associated with excellent barrier properties, increasing the effectiveness of the prevention of the permeation of gases such as oxygen or water steam [63]. Furthermore, a high E′ is beneficial during the manufacturing process because it ensures the material can be processed at elevated temperatures without compromising the mechanical properties of the final product. Therefore, heat-treated PLA/GrO composites have good application potential at a working temperature of less than 100 °C.

### 3.4. Thermogravimetric Analysis

The GrO addition influenced the rheological, mechanical, and thermal properties of the PLA/GrO composite. Figure S8 displays the TGA curves and their first derivative (DTG) of neat PLA and PLA/GrO composites. The TGA curves of G325 and GrO are shown in Figure S6. The neat PLA and PLA/GrO composites had a single thermal degradation starting at 280 °C and ending at 390 °C; see Figure S8a. The degradation temperature at 5%, 10%, and 50% weight loss, T_5%_, T_10%_, and T_50%_, respectively, of the PLA/GrO composites compared with neat PLA was analyzed. At T_5%_, the thermal stability of the PLA matrix diminished with GrO content, except for PLA/0.1GrO; see Figure S9. For example, PLA/3GrO was 11 °C lower than the neat PLA, and PLA/0.1GrO rose by 1 °C. Nevertheless, at T_10%_, neat PLA and PLA/0.1GrO had similar values. The temperature difference between neat PLA and PLA/0.1GrO was 9 °C, which was the lowest temperature of the PLA/GrO composites; see Figure S9. The decomposition temperatures at T_50%_ (Figure S10) for neat PLA, 0.1, 0.5, 1.0, 1.5, and 3.0 wt % PLA/GrO composites were 346.5 °C, 345 °C, 344 °C, 343 °C, 345 °C, and 345.5 °C, respectively. Chieng et al. reported a T_50%_ of 339.2 °C, 349.7 °C, and 339.7 °C for PLA, PLA/reduced graphene oxide (0.3 wt%), and PLA/graphene nanoplatelets (0.3 wt%), respectively [64]. Likewise, Wang et al. found a T_50%_ of 354.4 and 353.3 for PLA and PLA-starch/0.1 functionalized GO, respectively [65]. In addition, a decrease in the thermal degradation temperature (T_onset_) was observed in PLA/spent coffee ground (SCG) systems, where T_onset_ decreased as filler content increased [34].

The temperature corresponding to the maximum rate of weight loss (T_max_) was also analyzed (Figure S8). The T_max_ of the neat PLA, PLA/0.1GrO, PLA/0.5GrO, PLA/1GrO, PLA/1.5GrO, and PLA/3GrO was 349 °C, 348 °C, 350 °C, 347 °C, 352 °C, and 349 °C, respectively. According to these results, the temperature displacement value resulting from the difference in T_max_ PLA and T_max_ composites was ± 2–3 °C. Therefore, the T_max_ had no significant displacement values that indicate an improvement in the thermal stability of PLA/GrO composites concerning neat PLA. However, Valapa et al. [66] report an improvement in the thermal stability of PLA (T_max_ = 345 °C) when it contains 0.5 wt% graphene (T_max_ = 347 °C) in the polymer matrix. According to Bao et al. [67], the mass barrier effect becomes dominant when the graphene content is high enough for PLA/graphene composites, which results in a reduction in the maximum values in the DTG results. Additionally, a high thermal conductivity contributes to lower degradation temperatures.

### 3.5. Differential Scanning Calorimetry

The crystallization behavior and thermal properties of PLA can be modified with the inclusion of additives or fillers [68]. The DSC second heat treatment thermographs of neat PLA and PLA/GrO composites are shown in Figure S11. From these thermograms, the glass transition (T_g_), melting (T_m_), cold crystallization enthalpy (ΔH_cc_), fusion enthalpy (ΔH_f_), and cold crystallization (T_cc_) temperatures and crystallinity degree (χ_cc_) were determined; see Table 2.

The crystallinity degree (χ_cc_) was determined with Equation (1):

(1) $$\chi_{cc}=\frac{∆H_{cc}}{∆H_{100\%c}\left(1-\frac{\%wtGO}{100}\right)}\times 100$$

where $∆H_{100\%c}$, the fusion enthalpy of 100% crystalline PLA, is considered to be 93 J/g [69].

When the concentration of GrO in the PLA matrix increased, there was a slight decrease at first and then an enhancement in PLA T_g_, reaching the lowest temperature at 58 °C with a GrO concentration of 1 wt%, suggesting that the mobility of the PLA polymeric chains starts at lower temperatures and could be a consequence of the lubricant nature of the graphite. Similar behavior has been observed in PLA/spent coffee ground composites, where T_g_ values decrease as the filler content increases; this trend was observed by DSC and DMA and attributed to the increase in filler content [34,55].

Neat PLA presented a slight exothermic signal assigned to T_cc_ at around 125 °C. Table 2 displays the temperature corresponding to the T_cc_ of the composites. At about ΔH_cc_, the presence of GrO in the PLA matrix induced the formation of crystals, reaching the highest value at a concentration of 1 wt%. It is important to note that PLA/1GrO exhibited a higher E′_80°C,_ correlating with the crystal formation in the polymer matrix. Up to this concentration, the movement of the polymeric chains is allowed, so the formation of crystals is favored. This is similar to the DMA results, where E′ increases with GrO concentrations up to 1 wt%.

In contrast, higher GrO concentrations restricted the molecular mobility, hindering crystal formation, and ΔH_cc_ began to decrease. Therefore, there was less mechanical reinforcement as a consequence of cold crystallization achieved during heat treatment. The same behavior has been previously observed in PLA/Talc systems [28,29]. However, high particle concentrations led to improved physical interactions between the filler and the matrix [34,62], which is related to the increase in the slope of the time sweep curve of the PLA/3GrO sample (Figure 6a) due to the increase in the slope of the time sweep curve of the PLA/3GrO sample.

### 3.6. Transmission Electron Microscopy

**Figure 8:**
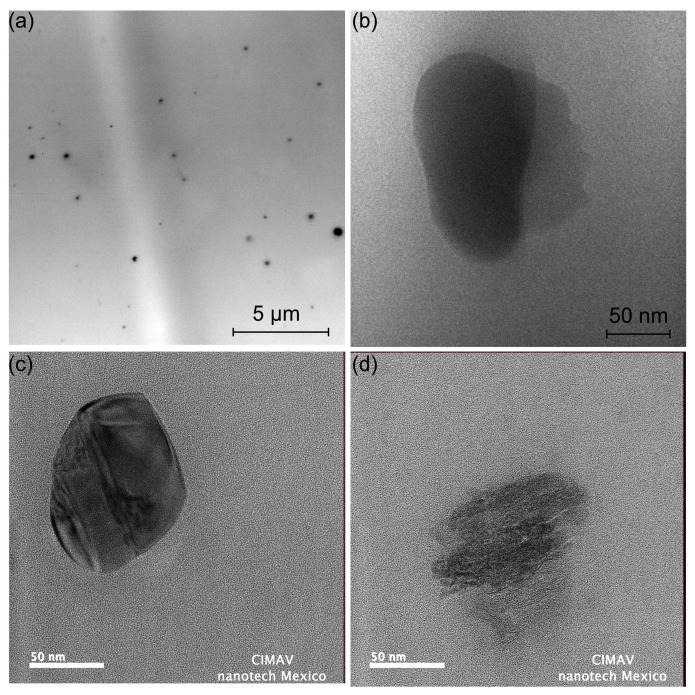
Transmission electron microscopy micrographs: (**a**) PLA/0.1GrO; (**b**) PLA/1GrO; (**c**) PLA/3GrO; (**d**) PLA/3GrO.

Figure 8 illustrates the TEM micrographs of the PLA/0.1GrO, PLA/1GrO, and PLA/3GrO composites. Figure 8a demonstrates that GrO particles were heterogeneously distributed in the PLA matrix, which was due to the method of incorporating the GrO particles into the PLA melt mix. Figure 8b illustrates a TEM micrograph of the PLA/1GrO composite. Compared with the GrO micrographs (Figure S1), particles with a nanometer size can be observed, suggesting the non-uniformity of sizes within the PLA matrix. Likewise, quasi-spherical nanometer particles were observed for PLA/3GrO in high-resolution TEM (Figure 8c,d). It has been reported that the formation of polymer chains relies on effective interactions between nanoparticles and the polymer substrate when employing the melt mixing technique. However, this method has its limitations in the production of nanocomposites. Among the more significant challenges is the occurrence of agglomerates or cavities in the field, along with the difficulty of achieving uniform distribution for particles with diverse morphologies [70,71]. Valapa et al. [66] studied the influence of temperature on the exfoliation of expandable graphite (EG) completely integrated into graphene (GR) sheets, and the solution was mixed with PLA at concentrations of 0.1, 0.3, and 0.5 wt% GR. They obtained a homogeneous distribution of GR sheets, with the formation of clusters of nanosheets within the PLA matrix. As the content of GR increased within the PLA matrix, the black portion of micrographs also increased. The observed outcomes may have resulted from the choice of mixing method utilized. The solution method is frequently employed and demonstrates effectiveness in promoting the mixing and dispersion of graphene. This is attributed to the solution’s low viscosity, which facilitates the process in the mixing system [70].

## 4. Conclusions

Polylactic acid (PLA)-based nanocomposites containing different concentrations of graphite oxide (GrO) were prepared by the melt mixing process, exploring the influence of elastic modulus when applying heat treatment on PLA/GrO composites. GrO underwent chemical structural alterations during melt blending while subjected to a temperature of 190 °C for 10 min. A new arrangement resulted from a change in the proportion of functional groups. The PLA matrix may have undergone more substantial degradation due to a more intense hydrolysis reaction as a result of this rearrangement. Additionally, the modification in GrO during processing caused a decrease in the thermal and rheological properties of the PLA/GrO composites, which was more evident at high GrO concentrations.

According to the TGA results, the temperature difference between neat PLA and PLA/GrO composites was ±2–3 °C. Therefore, T_max_ did not exhibit significant displacement values, indicating an improvement in the thermal stability of PLA/GrO composites relative to neat PLA. Regarding DSC, the crystallinity of PLA/GrO compounds was enhanced as a function of increased GrO concentration, reaching its maximum at 1 wt%. Compared with that of neat PLA, PLA/1GrO’s crystallinity increased by 57%.

PLA/GrO composites with GrO concentrations lower than 1 wt% showed enhanced thermomechanical properties in a glassy state. However, higher GrO concentrations presented a decrease in the elastic modulus. The elastic modulus (at 80 °C) of the PLA/GrO composites was affected by heat treatment at 110 °C for 30 min. PLA/1GrO (GrO concentration of 1 wt%) achieved a maximum increase in the elastic modulus of 4.62 × 10^8^ Pa. Up to this concentration, the movement of the PLA polymer chains was initiated around T_g_, possibly due to the lubricating properties of GrO, favoring the formation of crystals. On the contrary, above this GrO particle concentration, a reduction in crystallinity occurred due to the constrained movement of the polymer chains caused by the presence of GrO particles.

Future research will focus on increasing crystal formation and, therefore, the elastic modulus in the rubbery region by analyzing temperature and time using time sweeps in DMA.

## Figures and Tables

**Table 1:** XPS data of GrO and GrOmb (GrO treated under melt-blended conditions).

Sample		Peak BE (eV)	Bond	At %	C/O
GrO					2.4
	C1s	284.16	C=C sp^2^	0.0	
		285.13	C-C sp^3^	48.81	
		285.19	C-O	33.33	
		288.21	C=O	14.66	
		289.83	-COOH	3.18	
GrOmb					2.36
	C1s	284.16	C=C sp^2^	1.96	
		284.9	C-C sp^3^	59.3	
		286.99	C-O	21.55	
		287.86	C=O	17.18	
		289.83	-COOH	---	

**Table 2:** DSC results (second heating).

Sample	T_g_	T_cc_	T_m_	ΔH_cc_	ΔH_f_	χ_cc_
	°C	°C	°C	J/g	J/g	%
Neat PLA	60	125	145	10.4	10.5	11.1
PLA/0.1GrO	59	122	145	19.3	15.9	20.7
PLA/0.5GrO	59	119	149	20.5	18.2	22.1
PLA/1GrO	58	120	149	23.9	21.8	25.9
PLA/1.5GrO	58	128	151	14.9	13.9	16.2
PLA/3GrO	58	111	149	14.3	12.9	15.8

## Data Availability

The data presented in this study are available in the supplementary material.

## Supplementary Materials

The following supporting information can be downloaded at: https://www.mdpi.com/article/10.3390/polym16030431/s1. Figure S1: FE-SEM micrograph of (G325); (a) natural graphite flake micrographs of (G325); (b,c) oxidized graphite (GrO); (d, e, f); (g) GrO size distribution; Table S1: carbon and oxygen content according to EDS; Figure S2: Raman spectra of G325 and GrO; Figure S3: FTIR spectra of G325 and GrO; Figure S4: X-ray diffraction patterns of G325 and GrO; Figure S5: C_1_(s) XPS spectra of (a) G325 and (b) GrO; Figure S6: thermograms of G325 and GrO samples; Figure S7: E′ of neat PLA and PLA/GrO composites as a function of temperature; Figure S8: thermograms of neat PLA and PLA/GrO composites: (a) weight (%); (b) der. weight; Figure S9: thermograms of neat PLA and PLA/GrO composites at a loss weight of 5 and 10%.; Figure S10: thermograms of neat PLA and PLA/GrO composites at a loss weight of 50%. Figure S11: DSC thermograms of neat PLA and PLA/GrO composites. References [72,73,74,75,76,77,78,79,80,81,82,83,84,85,86,87] are cited in the supplementary materials.
