# Supplementary material for: Comprehensive Analysis of Rheological, Mechanical, and Thermal Properties in Poly(lactic acid)/Oxidized Graphite Composites: Exploring the Effect of Heat Treatment on Elastic Modulus
Source: Polymers (Basel). 2024 Feb 4;16(3):431. doi: 10.3390/polym16030431 (PMC10856878; doi:10.3390/polym16030431)
Supplement: Supplementary file 1 [file polymers-16-00431-s001.zip › polymers-2752730-supplementary.pdf]

Comprehensive Analysis of Rheological, Mechanical, and Thermal Properties in Poly (lactic acid)/Oxidized Graphite Composites: Exploring the Effect of Heat Treatment on Elastic Modulus

Mónica Elvira Mendoza-Duarte * and Alejandro Vega-Rios *

Centro de Investigación en Materiales Avanzados, S.C. (CIMAV), Av. Miguel de Cervantes #120, Complejo Industrial Chihuahua, Chihuahua 31136, Mexico
* Correspondence: monica.mendoza@cimav.edu.mx (M.E.M.-D.); alejandro.vega@cimav.edu.mx (A.V.-R.); Tel.: +52-614-4394831 (A.V.-R.)

S1. Characterization of G325 and GrO

S1.1 Field Emission Scanning Electron Microscopy

The FE-SEM micrographs of G325 and GrO at different magnifications are presented in Figure S1. G325, Figure S1(a, b, c) illustrates an orderly, flat, compact, and smooth structure. On the other hand, GrO (Figure S1 d, e, f) possesses a layered structure, which affords ultrathin and homogeneous graphene films. Such films are folded or continuous at times, and it is possible to distinguish the edges of individual sheets, including kinked and wrinkled areas.

The effect of the oxidation process over the G325 is more evident in FESEM. It is observed that G325 layers (Figure S1c) show a flat surface, whereas the GrO layers (Figure 1d, e, f) tend to wrinkle due to intercalation of the oxygen-containing functional groups as a consequence of the functional groups adhered to their surface during the oxidation process [72]. The corrugations and waves present in GrO give a higher thermodynamic stability, according to Carlsson [73] and Zhang [74].

Furthermore, GrO sheets show higher transparency, indicating a higher separation in the c-axis due to the functional groups adhering to their surface during the oxidation process [75].

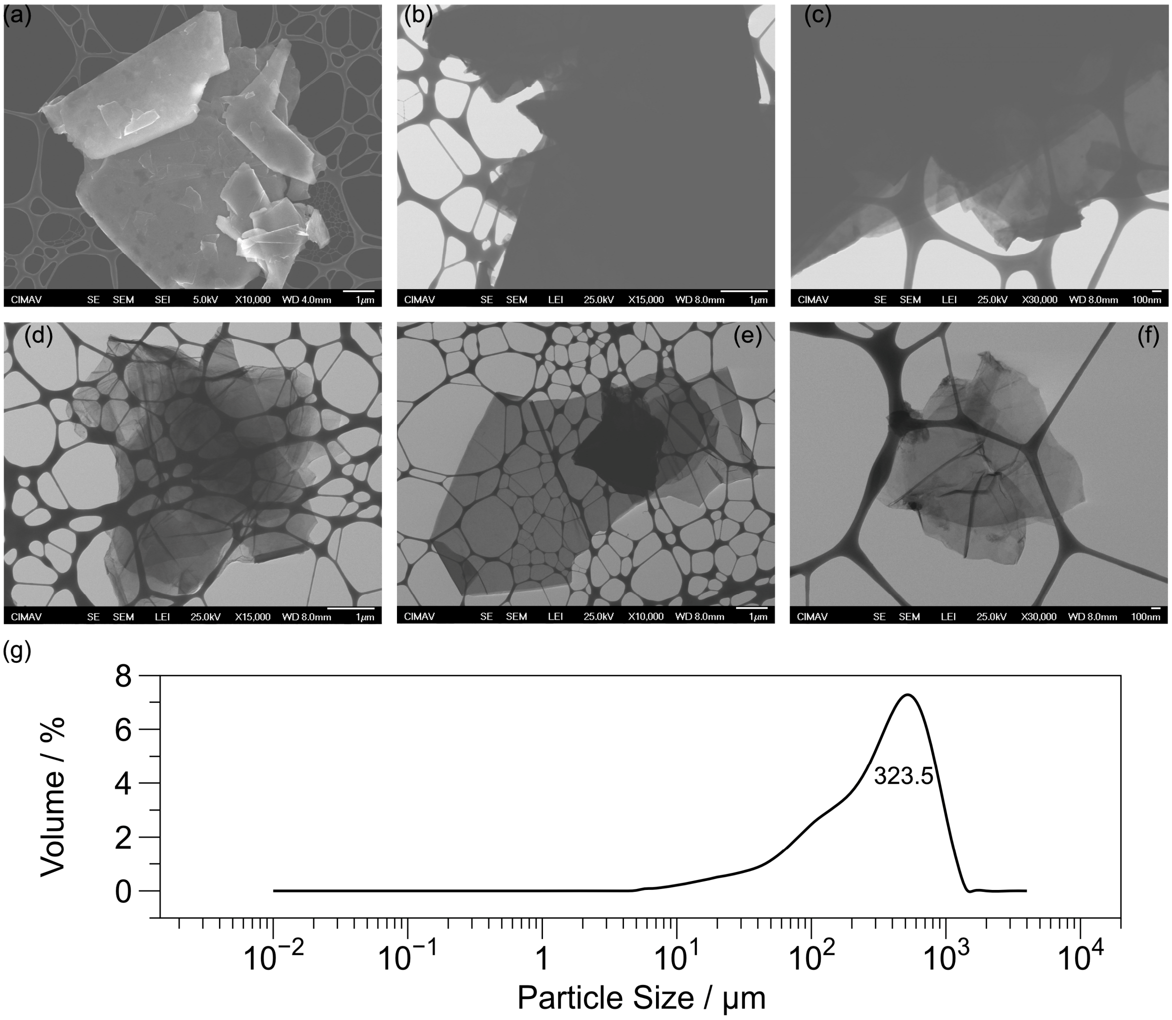

Figure S1. FE-SEM micrograph of (G325) (a) natural graphite flakes micrographs of (G325) (b,c) oxidized graphite (GrO) (d, e, f); (g) GrO size distribution.

S1.2 Energy Dispersive Spectra

To evaluate the content of the oxygen attached to the graphite surface during the oxidation reaction, G325 and GrO were analyzed by energy dispersive spectra at different points. Table S1 presents EDS results in each sample. In the case of G325, a low oxygen quantity is present (2.92%); this is attributed by Boehm [75] to chemisorbed oxygen located in the graphite edges due to the edge site's reactivity. GrO has a mean C content of around 59.84% and an O content of 40.16%, giving a ratio of O/C = 0.67. This ratio is lower than that obtained by Feng [76] and Oh [77] who reported O/C ratios of 0.806 and 0.834, respectively. No extra elements or impurities were found in G325 or GrO samples.

Table S1. Carbon and Oxygen Content by EDS.

**Sample**	**C At %**	**O At %**	**Ratio O/C**
G325	97.08	2.92	0.03
GrO	59.84	40.16	0.67

S1.3 Raman spectroscopy

Raman spectroscopy is widely used for characterizing carbon products, mainly because conjugated and double carbon-carbon bonds lead to high Raman intensities. For the carbon materials, the Raman spectra present a G signal around 1582 cm^{-1}, which is usually assigned to the C sp^2 atoms, and also shows a signal around 1350 cm^{-1}, which is related to a breathing mode of κ-point phonons of A1g symmetry.

The Raman spectra for G325 and GrO are presented in Figure S2. G325 spectra display a strong G line at 1582 cm^{-1}, a weak D signal at 1350 cm^{-1}, and a slightly broad D′ line at 2690 cm^{-1} [78]. For GrO, the G band is bonded and shifted to 1594 cm^{-1} whereas the D band at 1363 cm^{-1} becomes more prominent, indicating the creation of sp^3 domains due to the huge oxidation [79]. In addition, this increment in the D band is attributed to the significant decrease in the size of the in-plane sp^2 domains due to oxidation and ultrasonic exfoliation and the partially ordered graphite crystal structure of graphene nanosheets [78].

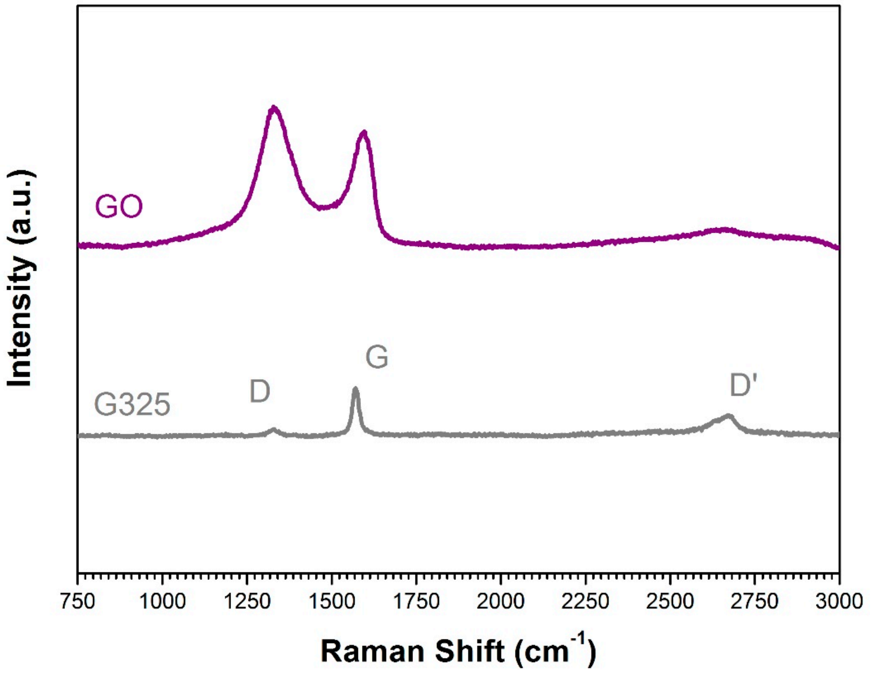

Figure S2. Raman spectra of G325 and GrO.

S1.4 Infrared Spectroscopy

The FT-IR spectra G325 and GrO are displayed in Figure S3. G325, no presence of any transmittance signal is observed, indicating that the graphite is free of functional groups. Conversely to G325, the spectrum of GrO presents several bands showing different functional groups attached to the graphene layers during the oxidation reaction. The signal around 3300 cm^{-1} corresponds to the O-H stretching of water molecules. The bands located between 1200 and 1631 cm^{-1} correspond to the absorbed H_2O molecules, carboxyl -OH stretching, and phenolic C-OH stretching [72].

The band at 1725 cm^{-1} in GrO is attributed to the C=O stretching vibrations of the carbonyl group [80]. The signal located at 1026 cm^{-1} is attributed to the C-O-C stretchings. The signal located at 937 cm^{-1} corresponds to the C-O stretch of the epoxy group [81].

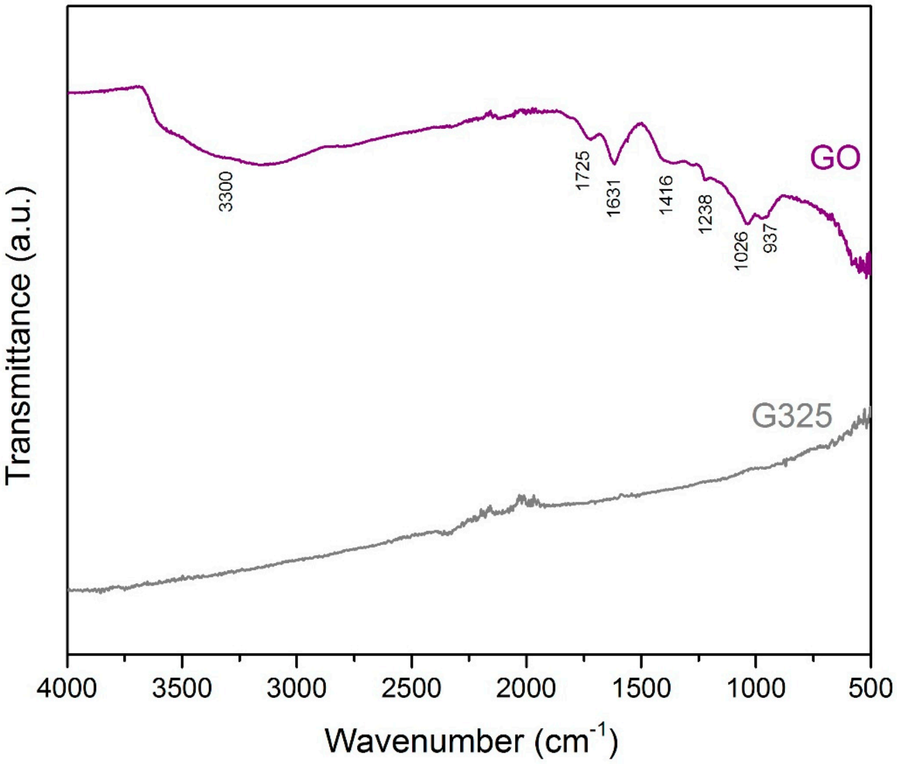

Figure S3. FTIR spectra of G325 and GrO.

S1.5 X-Ray Diffraction

XRD patterns of G325 a GrO are presented in Figure S4. G325 exhibits an intense signal at around 2θ ≈ 26.5° corresponding to the plane (002) and an interplanar distance of 0.34 nm, which is the distance between graphene sheets regularly stacked in graphite [82]. When the graphite is oxidized, GrO, this crystalline peak disappears, and a new signal appears around 2θ ≈ 11° corresponding to the (001) crystalline plane due to an increment of the interlayer distance from 0.34 nm to 0.846 nm attributed to the presence of water, carboxyl and hydroxyl groups attached to the graphene layers during oxidation process [82,83].

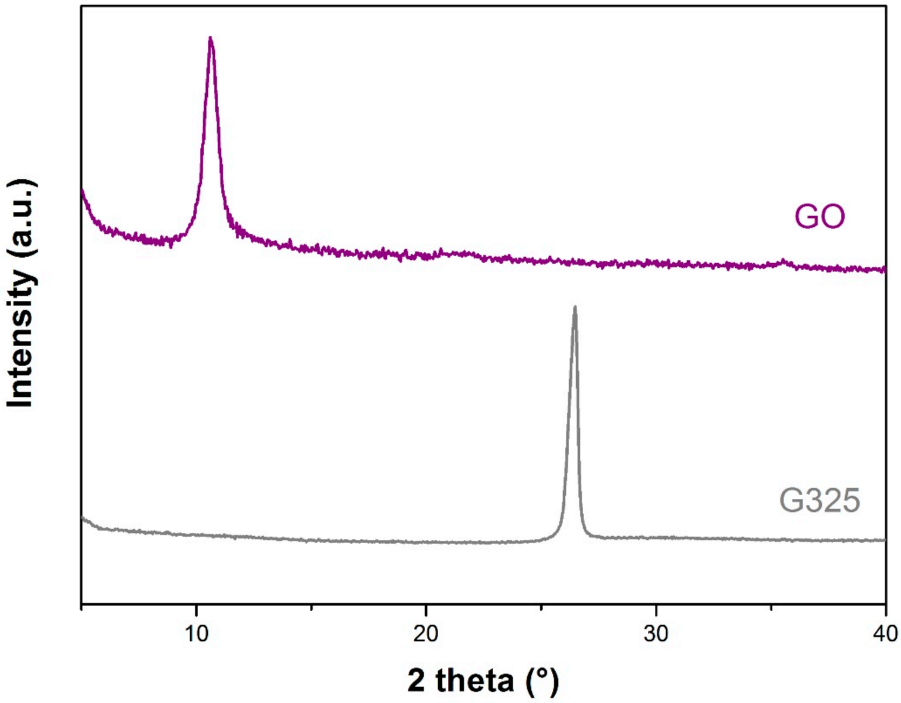

Figure S4. X-ray diffraction patterns of G325 and GrO.

S1.6 X-ray photo-electron spectroscopy

Figure S5 (a) and (b) show a comparison of the XPS C1s (284.8 eV) fitted spectra of pristine graphite (G325) and graphite oxide (GrO), respectively. GrO exhibits new signals attributed to the oxygen groups such as C-OR (287.19eV), C=O (288.21 eV) and -COO (289.93 eV).

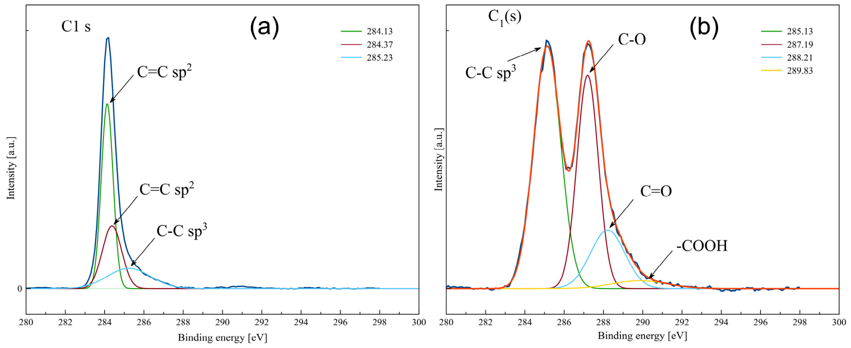

Figure S5. C_1(s) XPS spectra of (a) G325 and (b) GrO.

S1.7 Thermogravimetric analysis

The GrO displays two weight loss steps, Figure S6. A weight loss of around 10 % at the initial heating stage (50 °C-100 °C) corresponds to the removal of physically adsorbed water [84, 85]. The weight loss (10.52 %) between 142 °C and 192 °C is due to the decomposition of labile oxygen functional groups such as epoxy, carboxyl, and hydroxyl, the GrO [67, 86]. Afterward, the decomposition of the carbon structure started in the presence of the released oxygen from the functional groups, and this degradation continued till all the functional groups left the system [87].

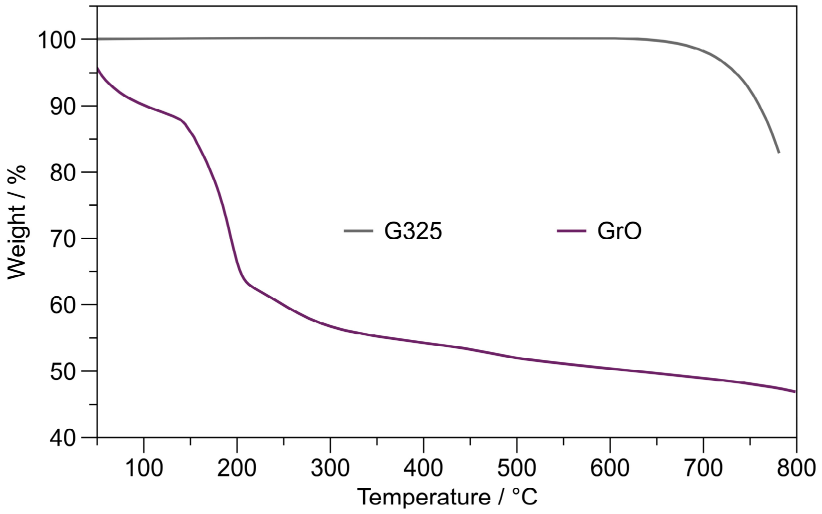

Figure S6. Thermograms of G325 and GrO samples.

S2. PLA/GrO Composites

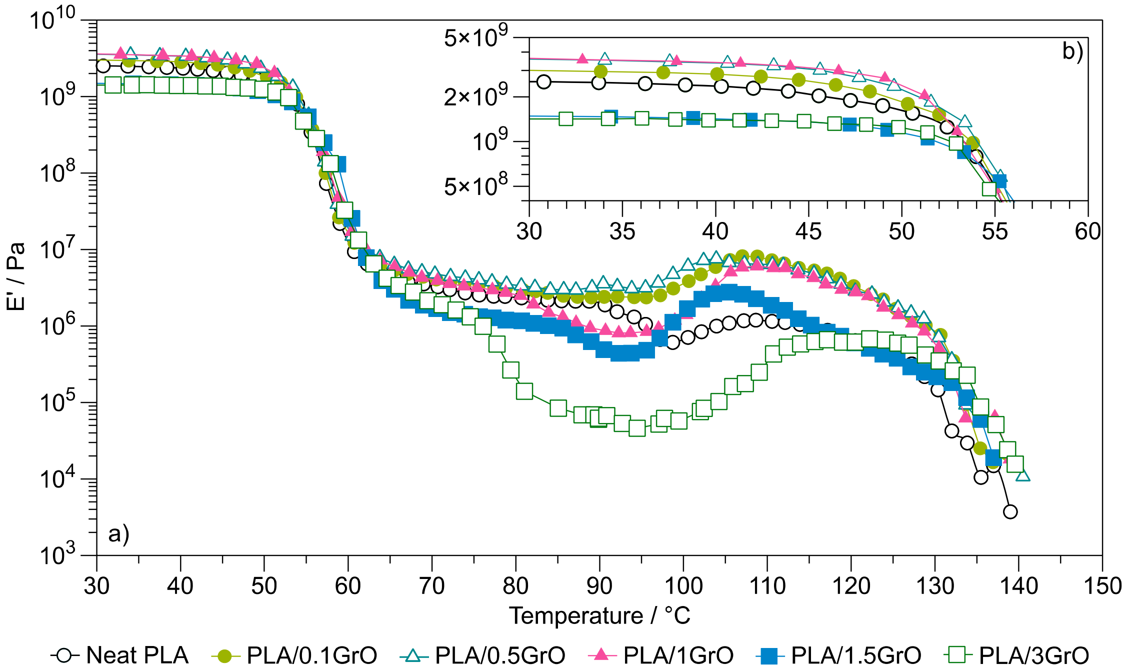

Figure S7. Storage modulus (E′) of neat PLA and PLA/GrO composites as a function of temperature.

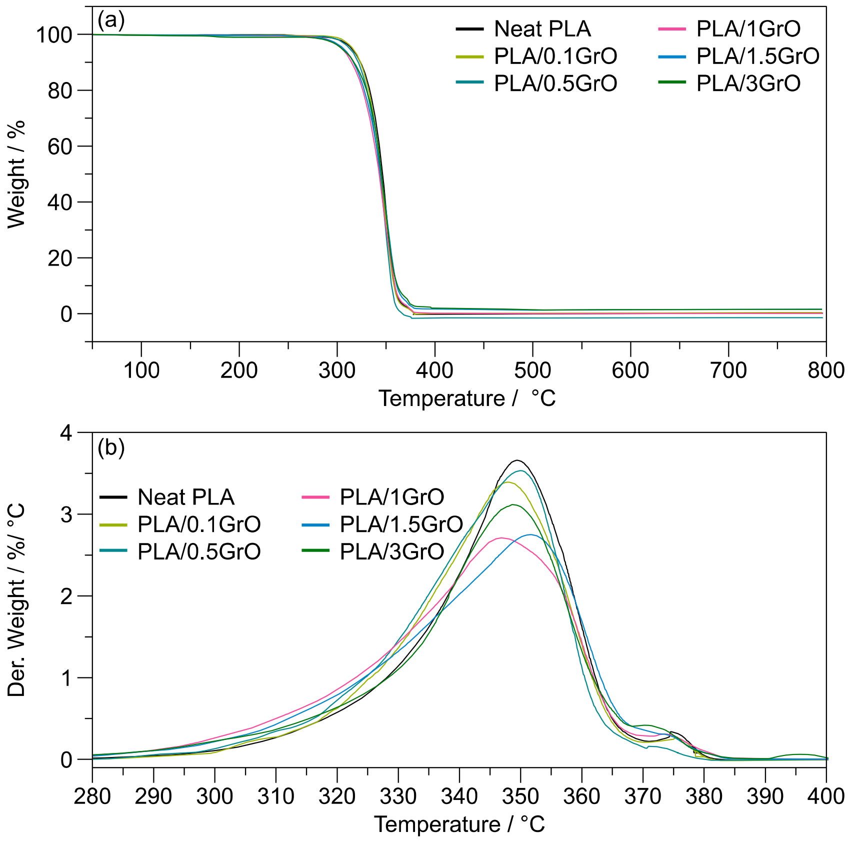

Figure S8. Thermograms of neat PLA and PLA/GrO composites. (a) Weight (%). (b) Der. Weight.

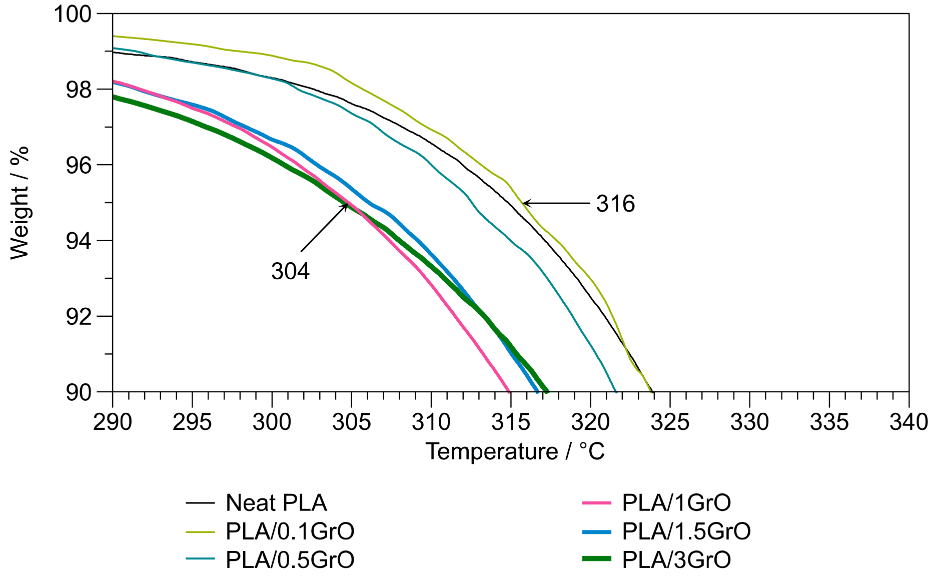

Figure S9. Thermograms of neat PLA and PLA/GrO composites at a loss weight of 5 and 10%.

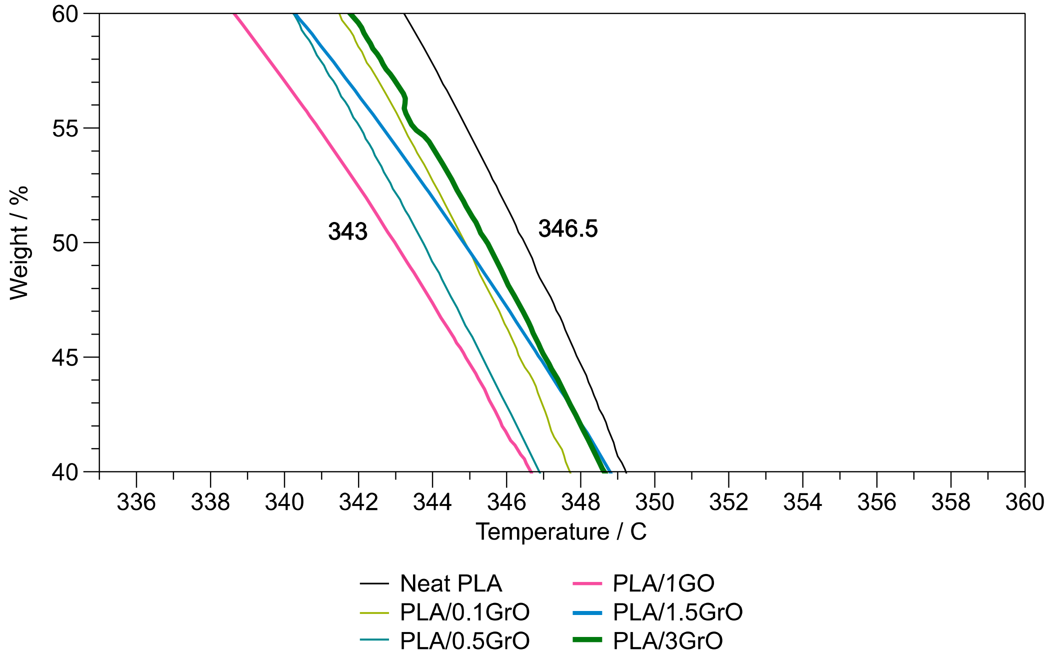

Figure S10. Thermograms of G325 and GrO at a loss weight of 50%.

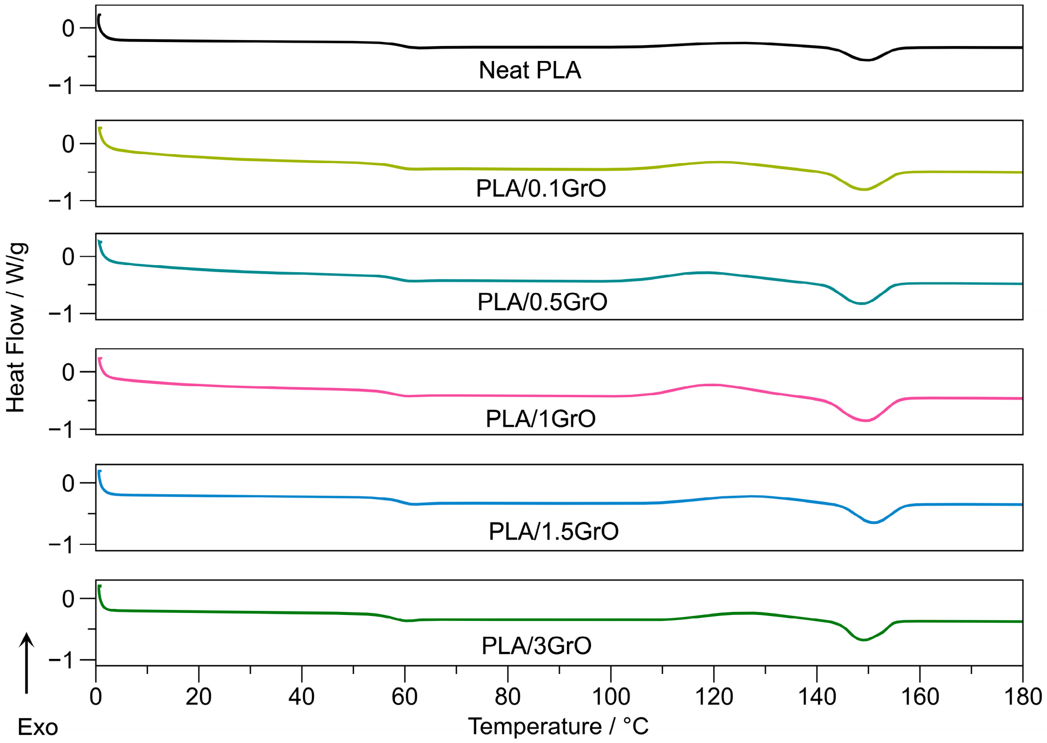

Figure S11. DSC thermograms of neat PLA and PLA/GrO composites.
